# Pure Squamous Cell Carcinoma of the Gallbladder Masquerading as a Hepatic Mass

**DOI:** 10.7759/cureus.2011

**Published:** 2018-01-01

**Authors:** Abhilash Perisetti, Saikiran Raghavapuram, Benjamin Tharian, Irfan Warraich, Fred Hardwicke, Rubayat Rahman, Edwin Onkendi

**Affiliations:** 1 Department of Hospital Medicine, Texas Tech University Health Sciences Center; 2 Division of Gastroenterology, University of Arkansas for Medical Sciences; 3 Department of Pathology, Texas Tech University Health Sciences Center; 4 Department of Hematology & Oncology, Texas Tech University Health Sciences Center; 5 Department of Surgery, Texas Tech University Health Sciences Center

**Keywords:** gall bladder, squamous cell cancer, liver tumors

## Abstract

Gallbladder (GB) carcinomas are adenocarcinomas (AC) in the majority of cases. Adenosquamous carcinoma (ASC) and pure squamous cell carcinoma (SCC) of the gallbladder are rarely encountered and comprise 1-3% of gallbladder cancer cases.  Pure squamous cell carcinoma of the gallbladder is rarer with less than 1% of the incidence. Most of the published literature is based on case reports and case series. The survival rates of ASC and SCC of the gallbladder are significantly lower (mean of five months) compared to the AC of the gallbladder (mean survival of 11.4 months). Most of these lesions are advanced at presentation, rendering them unresectable and resulting in a poor prognosis. However, if the lesions are diagnosed at an early stage, they could potentially be resectable. We report one such rare case of pure SCC GB presenting as a hepatic mass. The patient subsequently underwent resection of the gallbladder and liver mass with complete recovery and is currently planned for chemotherapy and radiation treatment.

## Introduction

Gallbladder carcinoma is an uncommon type of cancer of the gastrointestinal tract with a poor prognosis. It is diagnosed incidentally due to the vague symptoms. Adenocarcinoma (AC) remains the most common type of gallbladder cancer, followed by adenosquamous carcinoma (ASC) and squamous cell carcinoma (SCC). Most of these carcinomas are at the advanced stage at presentation, which precludes surgical resection as a treatment option. SCC of the gallbladder is an extremely rare type of cancer with a bulky appearance at presentation. If diagnosed early, surgical resection could potentially provide curative treatment. Surgery remains the mainstay of treatment for localized tumors with possible adjuvant chemotherapy and radiation as a further treatment option.

## Case presentation

A 68-year-old Hispanic male presented to the hospital with complaints of right upper abdominal pain, anorexia, nausea, and vomiting since one month. He had associated significant weight loss of more than 30 pounds over four weeks. No other associated symptoms were noted. Past medical history was unremarkable. No previous abdominal operations or prior history of alcohol, tobacco, or illicit drug use were noted. Vital signs were normal. On physical examination, he was in mild discomfort due to pain. He had no icterus. The abdomen was tender in the right upper quadrant. The anorectal exam showed no masses. Laboratory testing showed a white cell count of 13,400 per cubic milliliter, a hemoglobin of 14.8 mg per/dL, and an INR of 1.34 with normal liver function tests, including aspartate transaminase (AST), alanine aminotransaminase (ALT), total bilirubin, and hepatitis panel.

**Figure 1:**
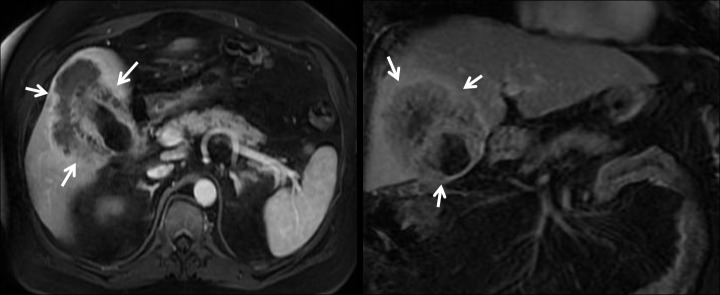
Magnetic resonance imaging (MRI) of the liver showing mass in relation (arrows) to portal vessels.

**Figure 2:**
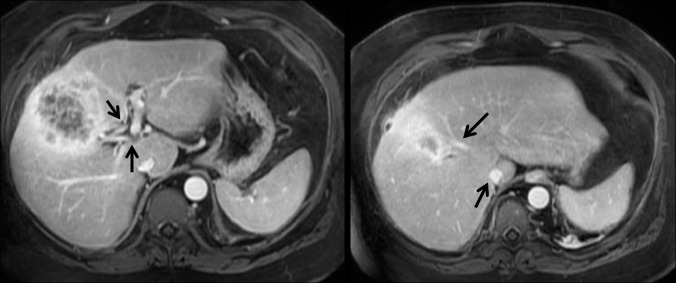
Magnetic resonance imaging (MRI) with no portal or hepatic vessel spread (arrows)

Ultrasonography (US) of the abdomen showed cholelithiasis with an ill-defined gallbladder wall contiguous with a complex hypoechoic mass in the adjacent right lobe of the liver measuring 8.7 x 8.8 cm in diameter. A computed tomography (CT) of the abdomen and pelvis with intravenous contrast showed a lobulated, multiloculated complex fluid collection with enhancing walls around the gallbladder fossa suggestive of a pericholecystic hepatic abscess measuring 6.8 cm in transverse, 8 cm anteroposterior, and 8.5 cm craniocaudally. The gallbladder wall was thickened, suggestive of acute cholecystitis. The common bile duct was 7 mm in size without evidence of any dilatation. A CT scan of the chest showed no masses in the lungs, mediastinum, or lymphadenopathy. An MRI of the abdomen was obtained to further characterize the liver and gallbladder changes, which ruled out additional lesions in the liver (Figures 1-2).

**Figure 3:**
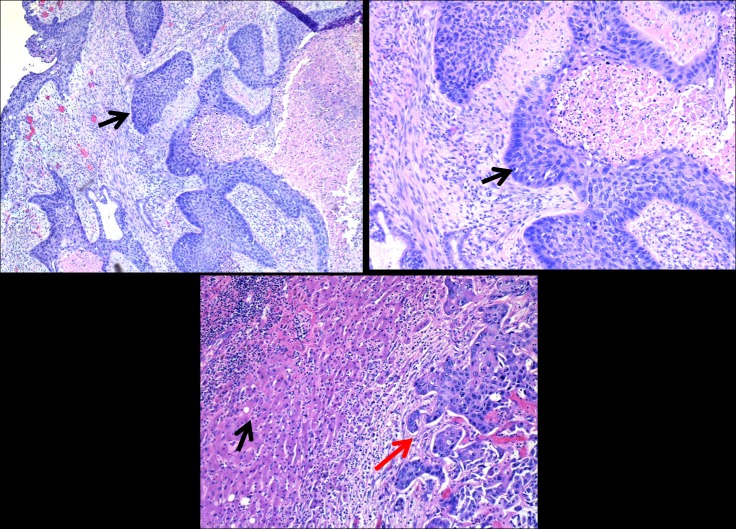
Ultrasound-guided core needle biopsy obtained from the hepatic mass Upper left: Liver squamous cell (SCC) islands with necrosis in the gallbladder wall; Upper right: mitotic figures and pleomorphism, hematoxylin and eosin (H&E) 50X; Lower panel: squamous cells islands (black arrow) in liver (red arrow), H&E 100X

It showed a large heterogeneous contrast-enhancing mass bridging segments IVa, IVb, and V of the liver and contiguous with thickening of the gallbladder wall involving the body and fundus. No other liver lesions were identified. There was no intrahepatic or extrahepatic biliary ductal dilatation seen or any specific evidence for metastatic disease. No direct involvement of the portal vein or the hepatoduodenal ligament. An ultrasound-guided core needle biopsy was obtained from the hepatic mass, and this showed the presence of invasive squamous cell carcinoma with the cells being p40-positive and focally CK7-positive. There were trapped bile ducts in many areas, but no convincing glandular component to the tumor (Figure 3).

**Figure 4:**
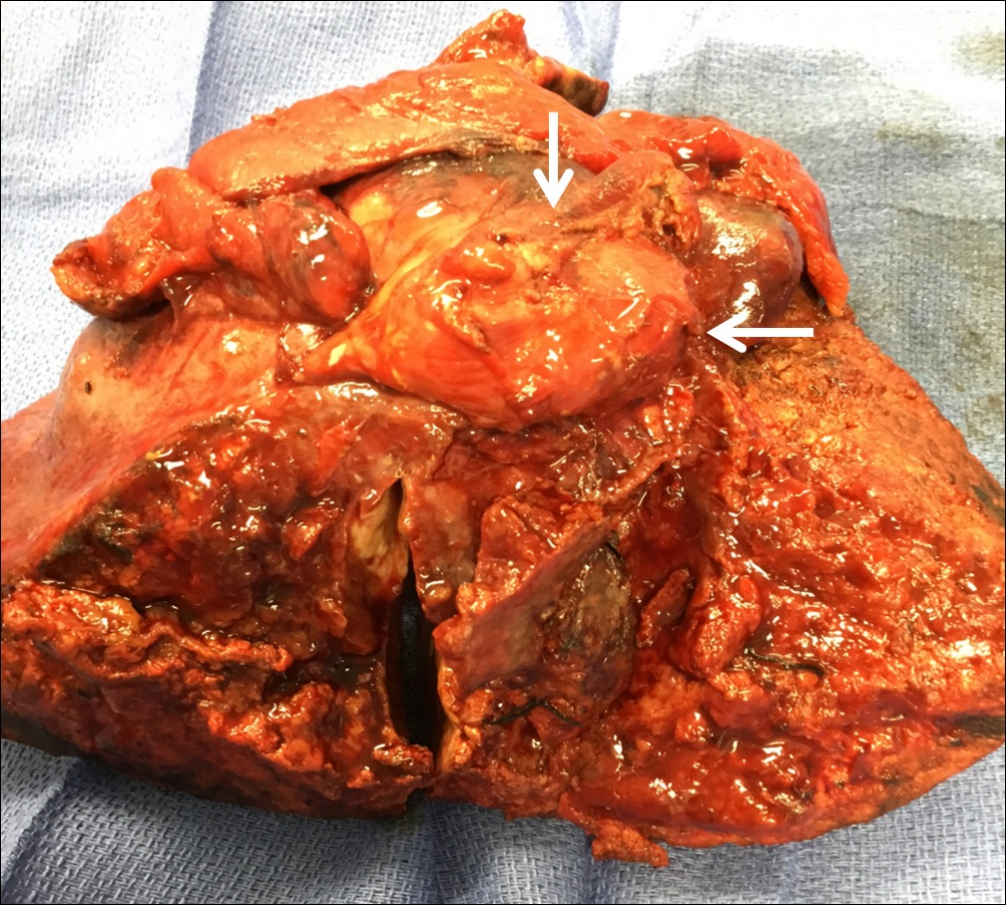
Resected gallbladder mass

**Figure 5:**
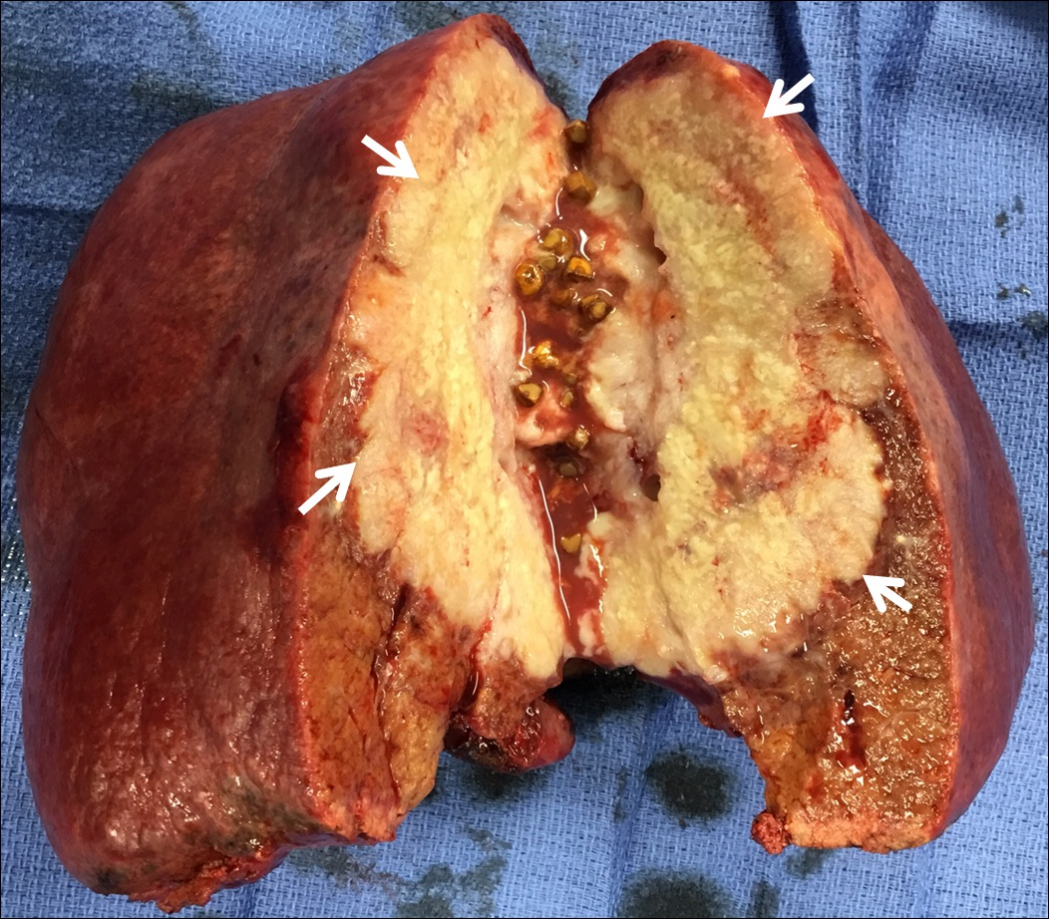
Liver mass (arrows) with resection of affected segments

Given the localized nature of the presumed gallbladder carcinoma with hepatic extension, curative resection was deemed possible. The patient underwent diagnostic laparoscopy, which was negative for carcinomatosis. A laparotomy with a central hepatectomy (segmentectomy Iva, IVb, V, and VIII) with en bloc cholecystectomy and hepatoduodenal lymphadenectomy were performed (Figures 4-5). 

Intraoperatively, there was no portal vein or omental involvement and no adjacent organ involvement. At the back table, the opened gallbladder showed multiple gallstones. Pathology showed pure squamous cell carcinoma of the gallbladder (without an adenomatous component) involving the gallbladder fundus to the neck with extensive invasion into the adjacent liver. The cystic duct margin was negative. The margins were tumor-free. All lymph nodes were negative. There was no angiolymphatic invasion. The patient recovered well after the surgery and has been scheduled for adjuvant chemoradiation therapy.

## Discussion

Squamous cell carcinoma (SCC) of the gallbladder is an extremely rare type of gallbladder carcinoma with a poor prognosis [1].  However, if identified at an early stage without distant spread, a potentially curative resection can be performed. In this case, SCC of the gallbladder showed extensive local invasion into the adjacent liver (segments IV and V) with no distant metastases. Gallbladder carcinomas can be divided into pure squamous cell carcinoma (SCC), adenosquamous (ASC), or adenocarcinoma (AC). Squamous differentiation is a term used when there is some component of squamous metaplasia with predominant adenomatous component [2]. Chronic irritation of the gallbladder due to gallstones increases the risk of gallbladder carcinoma [3], as was likely in this case given the multiple gallstones found. The gallbladder cancer incidence rates are reported to be higher in India, Pakistan, Ecuador, and Chile, with a female predominance (M:F - 1:3), likely due to a high incidence of cholelithiasis or other chronic gallbladder irritants. 

In 2011, Roa, et al. reported 41 cases with squamous differentiation out of 606 invasive gallbladder carcinomas [2], with eight cases of pure squamous cell carcinoma. Most of the cases are diagnosed intraoperatively, and rarely by endoscopic ultrasound with fine-needle aspiration [4-5]. Song, et al. reported 411 patients with gallbladder carcinoma, of which 377 patients had AC, 24 had ASC, and 10 had pure SCC [6]. The mean age was 61.4 years with a predominant association with gallstones. The ASC and SCC patients had a larger tumor burden, with noted liver involvement, compared to the AC type. Of these, the average age was 65 years, with a female preponderance and prominent keratinization. Survival of patients with ASC and SCC was significantly worse compared to adenocarcinoma. Risk factors for the squamous and non-squamous types of gallbladder carcinomas vary [7]. SCC of the gallbladder has an extremely poor prognosis with a mean survival of five months. However, early stage cases, while still localized, might be amenable to operative resection with improved survival. A rare association with parasitic infections has been noted, specifically, the biliary fluke. However, this is in very few cases and areas endemic to parasitic infection [8]. In our case, stool testing for liver fluke, schistosomiasis, and Entamoeba histolytica were all negative. Given the early detection and treatment of this patient, a potential cure was possible.

This case was initially presumed to be an acute gangrenous cholecystitis with rupture into the adjacent liver with abscess but was later diagnosed as a gallbladder carcinoma on intraoperative biopsy. Some of the gallbladder SCC cases reported local extension to the liver, extension to the porta hepatis with biliary obstruction, and hepatic flexure colonic involvement [9].  Compared with AC, SCCs of the gallbladder are bulky in nature with a high proliferative rate and a lower likelihood of lymph nodal metastases. Due to its bulky nature, these tumors are more likely to be symptomatic at early stages and, if localized, can be curatively resected. Our case was subjected to complete curative resection with tumor-free margins and had no lymph node involvement. Postoperative adjuvant chemotherapy and radiotherapy have been tried in few cases. Bourmeche, et al. reported the use of 45 Gy, combined with 5 fluorouracil and cisplatin chemotherapy, with complete remission [10]. Based on the current literature and review of few cases of pure SCC of GB, postoperative chemotherapy and radiotherapy could be the best therapeutic option. 

## Conclusions

In conclusion, we report an extremely rare case of SCC of the gallbladder presenting with an extensive local invasion of the adjacent liver. This case provides an example of surgical resection of a gallbladder SCC resulting in complete recovery with tumor-free margins, followed by chemotherapy and radiation with close surveillance. Gallbladder cancers traditionally carry a poor prognosis due to a late presentation and an advanced stage at diagnosis. However, if identified early, surgical resection could be potentially curative and offer a better prognosis.

## References

[1] Albores-Saavedra J, Henson DE, Sobin LH (1992). The WHO Histological Classification of Tumors of the Gallbladder and Extrahepatic Bile Ducts. A commentary on the second edition. Cancer.

[2] Roa JC, Tapia O, Cakir A (2011). Squamous cell and adenosquamous carcinomas of the gallbladder: clinicopathological analysis of 34 cases identified in 606 carcinomas. Mod Pathol.

[3] Randi G, Franceschi S, La Vecchia C (2006). Gallbladder cancer worldwide: geographical distribution and risk factors. Int J Cancer.

[4] Chambers MR, Hasan MK, Hébert-Magee S (2016). Pearls before bile: Primary squamous cell carcinoma of the gallbladder diagnosed on-site by endoscopic ultrasound-guided fine-needle aspiration.. Dig Endosc.

[5] Gupta P, Gupta RK (2012). Preoperative diagnosis of squamous cell carcinoma of the gallbladder by ultrasound-guided aspiration cytology: clinical and cytological findings of nine cases. J Gastrointest Cancer.

[6] Song HW, Chen C, Shen HX (2015). Squamous/adenosquamous carcinoma of the gallbladder: Analysis of 34 cases and comparison of clinicopathologic features and surgical outcomes with adenocarcinoma. J Surg Oncol.

[7] Andrea C, Francesco C (2003). Squamous-cell and non-squamous-cell carcinomas of the gallbladder have different risk factors. Lancet Oncol.

[8] Gómez N, Urrea I, Astudillio R (1990). Primary epidermoid carcinoma of the gallbladder. Acta Gastroenterol Latinoam.

[9] Mghirbi F, Ayadi M, Karray W (2016). Squamous cell carcinoma of the gallbladder. Transl Gastroenterol Hepatol.

[10] Bourmèche M, Ben Salah H, Kallel M (2013). A long survival after the treatment of a squamous cell carcinoma of the gallbladder (Article in French). Cancer Radiother.

